# Identifying Hub Genes and Immune Cell Infiltration for the Progression of Carotid Atherosclerotic Plaques in the Context of Predictive and Preventive Using Integrative Bioinformatics Approaches and Machine-Learning Strategies

**DOI:** 10.1155/2022/7657379

**Published:** 2022-10-18

**Authors:** Han Zhang, Yinde Huang, Xin Li, Wenbin Chen, Yu Lun, Jian Zhang

**Affiliations:** Department of Vascular and Thyroid Surgery, The First Affiliated Hospital of China Medical University, Shenyang, Liaoning 110001, China

## Abstract

Emerging evidence shows that carotid atherosclerosis is related to the activation of immune-related pathways and inflammatory cell infiltration. However, the immune-linked pathways that helped in the advancement of the carotid atherosclerotic plaque and the association of such plaques with the infiltration status of the body's immune cells still unclear. Here, the expression profiles of the genes expressed during the progression of the carotid atherosclerotic plaques were retrieved from the Gene Expression Omnibus database and 178 differentially expressed genes were examined. The Weighted Gene Coexpression Network Analysis technique identified one of the brown modules showed the greatest correlation with carotid atherosclerotic plaques. In total, 66 intersecting genes could be detected after combining the DEGs. LASSO regression analysis was subsequently performed to obtain five hub genes as potential biomarkers for carotid atherosclerotic plaques. The functional analysis emphasized the vital roles played by the inflammation- and immune system-related pathways in this disease. The immune cell infiltration results highlighted the significant correlation among the CD4+ T cells, B cells, macrophages, and CD8+ T cells. Thereafter, the gene expression levels and the diagnostic values related to every hub gene were further validated. The above results indicated that macrophages, B cells, CD4+ T cells, and CD8 + T cells were closely related to the formation of the advanced-stage carotid atherosclerotic plaques. Based on the results, it could be hypothesized that the expression of hub genes (C3AR1, SLAMF8, TMEM176A, FERMT3, and GIMAP4) assisted in the advancement of the early-stage to advanced-stage carotid atherosclerotic plaque through immune-related signaling pathways. This may help to provide novel strategies for the treatment of carotid plaque in the context of predictive, preventive, and personalized medicine.

## 1. Introduction

Carotid atherosclerosis, a chronic inflammatory arterial disease characterized by lipid accumulation in the walls of blood vessels, leads to the formation of atherosclerotic plaques. It is regarded as the primary trigger of cerebrovascular diseases [1]. Advanced-stage carotid plaques are very severe and can easily undergo rupture during carotid plaque development than early-stage carotid plaques [2]. The main cause of cerebrovascular events is the blood vessel blockage caused by plaque rupture, which results in thrombus formation in the carotid artery [3]. Inflammation, oxidative stress, and lipoprotein aggregation have all been implicated in the progression of the early-stage lesions into advanced-stage plaques [4, 5]. Plaque progression is also influenced by physical, hemodynamic, and biomechanical factors [6]. Immune and inflammatory responses play a vital role in all phases of atherosclerotic plaque progression [7]. Therefore, for early screening, intervention, and discovery of new therapeutic targets, it is essential to completely understand the inherent molecular mechanisms and immunological inflammation-related pathways linked to the onset and progression of carotid plaques.

Earlier reports have highlighted the factors responsible for the progression of the carotid atherosclerotic plaque. For instance, it has been observed that inflammatory cytokines like the Interleukins (IL)-1b, IL-6, IL-17, IL-8, and tumor necrosis factor-alpha (TNF-*α*), aid in the development of plaques [8]. One of the earlier studies showed that the regulatory T cells (Tregs) activate anti-inflammatory macrophages, suppress the formation of foam cells, and affect cholesterol metabolism, all of which have an atherosclerosis-preventing effect [9]. In contrast, macrophages, and neutrophil extracellular traps (NETs) were shown to promote inflammation and atherosclerotic plaque progression [10, 11]. Although prior research has shown that different immune cells and immune system-related pathways were involved in the progression of carotid atherosclerotic plaques, none of the reports have determined the major molecular processes and how they interact with the inflammation-linked pathways to control the progression of the carotid atherosclerotic plaques.

Based on the aforementioned findings, the goal of this work was to conduct a bioinformatics analysis to uncover vital biomarkers and immune-linked pathways involved in the development of the carotid atherosclerotic plaques. Furthermore, their interaction with the infiltration status of different immune cells was also studied. Two gene expression datasets, GSE28829 and GSE43292, were acquired from the Gene Expression Omnibus (GEO) database. After screening for the differentially expressed genes (DEGs), a Weighted Gene Coexpression Network Analysis (WGCNA) technique in combination with Least Absolute Shrinkage and Selection Operator (LASSO) technique, was implemented for screening the hub genes responsible for the progression of advanced-stage carotid atherosclerotic plaques. The functions of the screened DEGs were then determined by Gene Ontology (GO) and the Kyoto Encyclopedia of Genes and Genomes (KEGG) analyses. The correlation between immune cell infiltration and DEGs was then examined using the single-sample GSEA (ssGSEA) technique. The results of the above experimental procedures offer insightful information regarding the effect of inflammation-related pathways on the advancement of the carotid atherosclerotic plaques. Thus, this report could be used as a foundation for further investigation into the pathophysiology, targeted prevention, diagnostic procedures, and therapeutic targets of carotid atherosclerotic plaques.

## 2. Materials and Methods

### 2.1. Data Retrieval

A public database, GEO (http://www.ncbi.nlm.nih.gov/geo) [12], contains a variety of datasets related to the high-throughput gene sequencing and microarray data, submitted by different research institutions all over the world. The phrase ‘carotid atherosclerotic plaques' was used as a keyword in retrieving two related gene expression datasets. After the GEO database analysis, 2 datasets, GSE28829 [13] and GSE43292 [14], were chosen for further analyses. Hub genes were screened using the GSE28829 database, while the data was validated using the GSE43292 dataset. The GSE28829 dataset, which was sequenced on the GPL570 platform, consists of 13 samples of early-stage carotid atherosclerotic plaque and 16 samples of advanced-stage carotid atherosclerotic plaque. GPL6244 served as the sequencing platform for GSE43292, which contains 32 atheroma plaque samples and paired macroscopically intact tissues. The data we used in this study was obtained from online data repository and no experiment was performed.

### 2.2. Identifying the DEGs

The GSE28829 series matrix files were annotated with the GPL570 data table. To detect the DEGs between the early and advanced groups, gene expression patterns from each group were normalized and compared using the “limma” R program. The screening criteria for DEGs were set as adjusted P-value <0.05 and the |logFC (fold change) | ≥1.

### 2.3. Functional Enrichment Analysis

The “clusterProfiler” and the “enrichplot” packages of R were used for the GO analyses of DEGs. Metascape (http://metascape.org), an online analysis platform, was used to performed KEGG analyses [15], with *P* < 0.05 suggesting a significant difference.

### 2.4. Constructing the Gene Coexpression Network

A weighted coexpression network was developed with the help of a WGCNA package of R software for the expression profile data of the GSE28829 dataset. The genes that showed the highest 25% absolute deviation from their median values were then selected for further examination [16]. The R software's “limma” function was utilized for verifying the data integrity. The “pickSoftThreshold” function was implemented for choosing and verifying the optimal soft threshold (*β*) value. To identify the modules depending on their topological overlap, all matrix data were converted into the adjacency matrix and clustered. A hierarchical clustering dendrogram was created after computing the Module Eigengene (ME) and using the ME value for determining the similar merging modules in the clustering tree. For quantifying the significance of the genes and related clinical data, modules, and the phenotypic data were integrated to determine gene significance (GS) and module significance (MS) and to examine the relationship between modules and models. For every gene, the module membership (MM) was derived for assessing the GS values in the module.

### 2.5. Identification and Validation of All Identified Hub Genes

Candidate hub genes were chosen based on their highest intermodule connectivity. It was stated that the absolute GS values are usually higher in genes with a biological significance. The absolute values of GS>0.50 and MM>0.80 were applied as screening parameters for candidate hub genes. The DEGs and the candidate hub genes were then intersected with the aid of the R software's Venn diagram tool. The final set of hub genes was screened by LASSO regression analysis using the R software's “glmnet” package [17]. Box plots were applied for evaluating the expression levels of the hub genes in the patients with early- and advanced-stage carotid atherosclerotic plaques in the GSE28829 dataset. To evaluate the expression levels of the identified hub genes for differentiating between patients with early- and advanced-stage carotid atherosclerotic plaques, receiver operating characteristic (ROC) curves were generated using the “pROC” package in the R software. Another external dataset, GSE43292, was used for validating the hub genes' expression levels and diagnostic significance.

### 2.6. Assessment of Immune Cell Infiltration and Its Correlation with Hub Genes

The ssGSEA technique was applied to measure the relative levels of infiltration of 28 different immune cells using the GSE28829 dataset [18]. R software was used to generate heat maps and violin plots for demonstrating the differential expression levels of 28 infiltrating cells. The “ggplot2” software was utilized to visualize Spearman's correlation coefficients for the 28 immune-infiltrating cells with the hub genes.

## 3. Results

### 3.1. Constructing a Coexpression Network and Identifying the Hub Module

**Figure 1:**
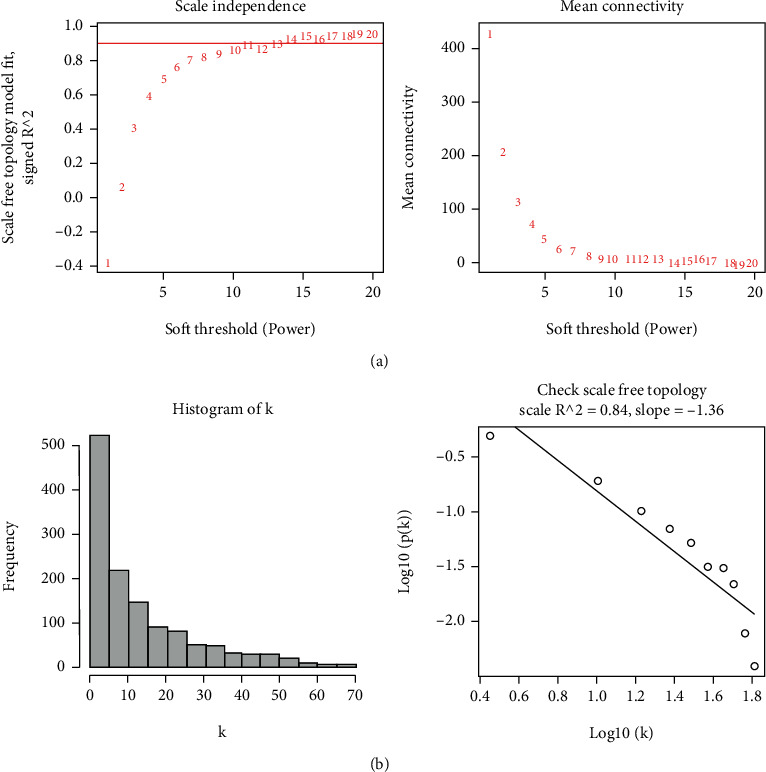
Estimating the soft-thresholding power in the WGCNA. (a) Analysis of a scale-free fit index and average connectivity for different soft-thresholding values (*β*). The red line denotes the region where the correlation coefficient is 0.9, and the respective soft-thresholding power is derived using the R software as 9. (b) Histogram indicating the connectivity distribution and determining the scale-free topology if *β* = 9.

Before processing missing values and eliminating outliers, samples were clustered and 1262 genes showing a top 25% absolute deviation from their median values were chosen for developing the WGCNA-based model. In order to ensure consistency with the scale-free network, the value of the soft threshold factor, *β*, was established as 4 (scale-free *R*^2^ = 0.84; slope = 1.36) (Figure 1).

**Figure 2:**
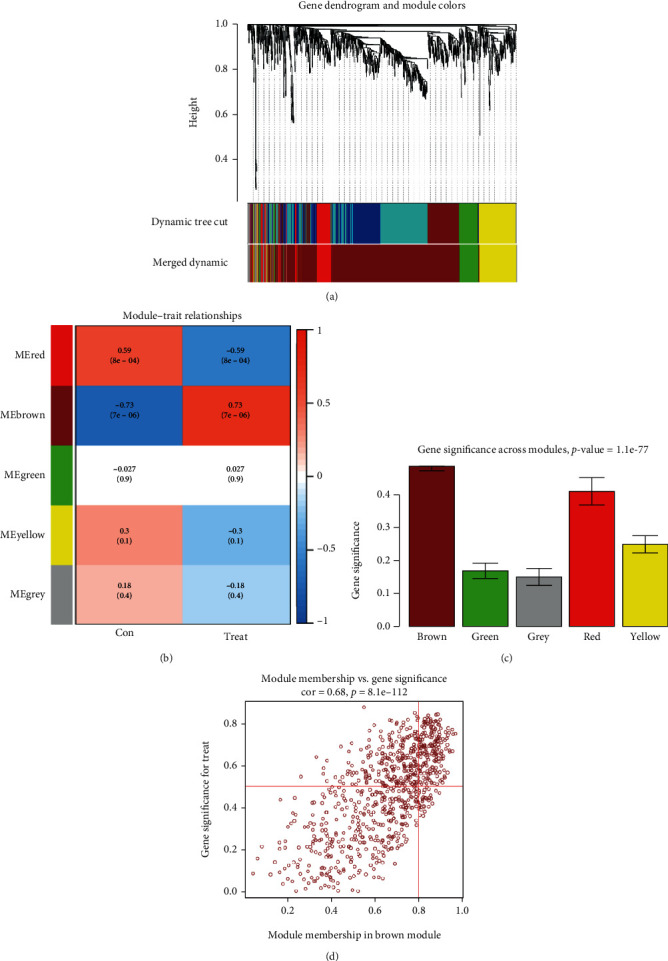
Constructing the WGCNA modules. (a) A cluster dendrogram of the genes having a median absolute deviation in the top 25%. Every branch denotes a single gene, while each color indicates a coexpression module. (b) Heat maps indicating the relationship between the module and trait. The brown module shows a significant relationship with the carotid atherosclerotic plaques. (c) Distribution of the mean gene significance in the modules associated with the carotid atherosclerotic plaques. (d) Scatter plots describing the correlation between the gene significance and gene module membership in the brown module. WGCNA: Weighted Gene Coexpression Network Analysis.

The network's coexpression matrix was then built using a 1-step technique, and five modules were generated using dynamic hybrid shearing (Figure 2(a)). Heat maps demonstrated the relationships between the aforementioned modules and advanced- and early-stage carotid atherosclerotic plaques. One hub module, i.e., the brown module, which contained 816 genes, had the strongest relationship (cor) with advanced-stage carotid atherosclerotic plaques (cor = 0.73, *p* = 7*e* − 6) (Figures 2(b) and 2(c)). Within the brown module, GS and MM also showed a strong association (cor = 0.68; *p* = 8.1*e* − 112) (Figure 2(d)).

Hence, the brown module was chosen as a vital module for additional analysis. Based on the above screening criteria, 195 genes that displayed the maximum connectivity in this brown module were chosen as candidate hub genes (absolute GS value>0.50; absolute MM value>0.80).

### 3.2. Identifying the DEGs and Screening the Hub Genes

**Figure 3:**
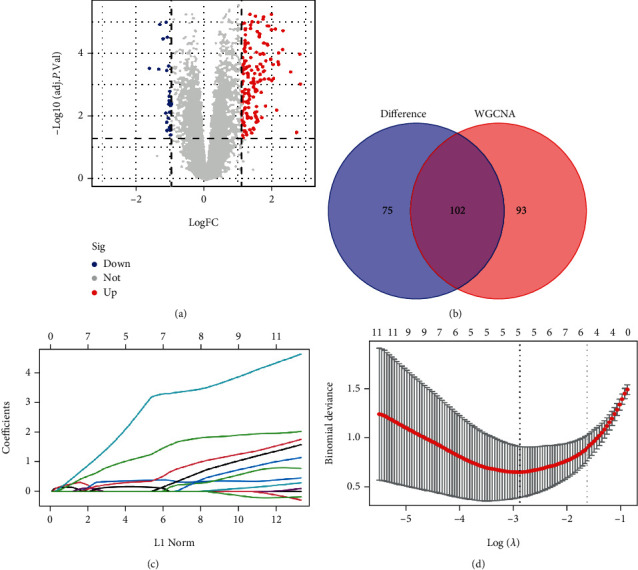
Identifying the DEGs and the sreening of hub genes. (a) Volcano plots for the DEGs in the early and advanced carotid atherosclerotic plaques. (b) Venn diagram indicating the intersection between the brown module and DEGs. (c) Partial likelihood deviance having a changing of log (*λ*) that was plotted using the LASSO regression in the 10-fold cross-validation. The dotted vertical lines were plotted using the minimal criteria (lambda.min) and 1-SE of minimal criteria (1-SE criteria) at the optimal values. (d) LASSO coefficient profiles of the 5 hub genes using 10-fold cross-validation. DEGs: Differentially Expressed Genes. LASSO: Least Absolute Shrinkage and Selection Operator.

Using an adjusted *P* value <0.05 and a |logFC (fold change) | ≥ 1, 177 DEGs in the GSE28829 dataset were detected, which included 154 upregulated and 23 downregulated genes.  Figure 3(a) presents the volcano plots for the DEGs. Thereafter, 102 intersecting genes were acquired based on the intersection in the Venn diagram (Figure 3(b)). Using LASSO analysis, five hub genes were discovered, including the signaling lymphocyte activating molecule family 8 (*SLAMF8*), complement C3a receptor 1 (*C3AR1*), FERM domain-containing kindlin 3 (*FERMT3*), transmembrane protein 176A (*TMEM176A*), and GTPases of the immunity-associated protein (*GIMAP4*) (Figures 3(c) and 3(d)).

### 3.3. Functional Enrichment Analysis of DEGs

**Figure 4:**
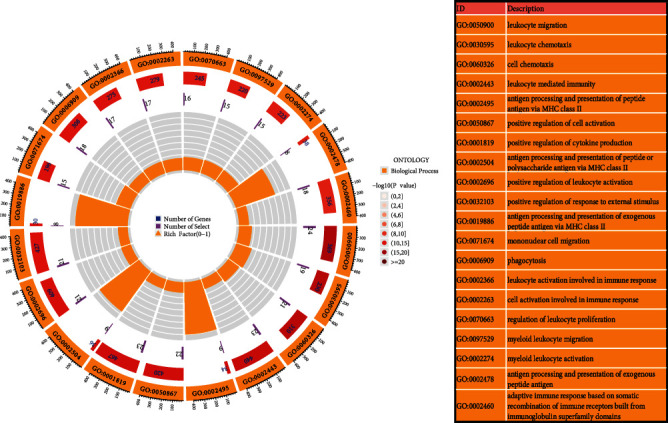
GO analysis of the DEGs in different biological processes. Lap 1 indicates the top 20 GO terms, while the number of genes is listed in the outer lap. Lap 2 shows the number of background genes in the human genome as well as *P* values for DEG enrichment for certain biological processes. Lap 3 presents the number of genes that are enriched in this process. Lap 4 denotes the enrichment factor for each GO term. GO: Gene Ontology.

After conversion into the gene ID, the GO and KEGG pathway enrichment analyses were carried out for analyzing the biological roles and pathways that the 177 DEGs were related to in the advanced-stage carotid atherosclerotic plaques. The GO annotations of DEGs comprised three parts, i.e., biological processes (BP), cellular components, and molecular functions, which were used to analyze the functional enrichment of DEGs. With respect to BPs, the GO enrichment analysis revealed that the identified DEGs were primarily enriched in many defense processes (such as antigen processing and presentation, cell migration, cell activation, leukocyte activation, and cell chemotaxis) and the immune and inflammation-related pathways (i.e., leukocyte mediated immunity, cytokine production, and immune response) (Figure 4).

**Figure 5:**
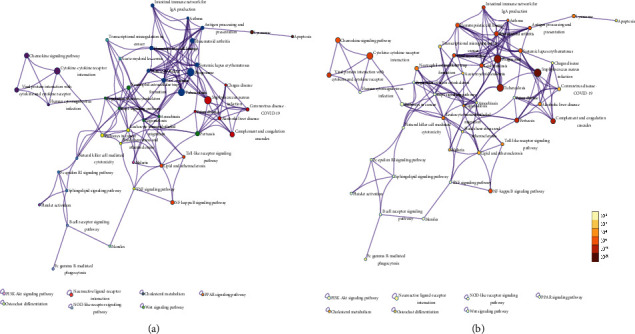
KEGG enrichment analysis of the DEGs in different biological processes. Figure 5 shows the KEGG pathway enrichment analysis findings. (a) Cluster IDs are color-coded, with nodes that share the same cluster ID being close to each other; (b) *P* values are colored according to the number of genes they contain, where terms with a higher p-value tend to be more significant.

With respect to the KEGG pathway, the identified DEGs were primarily enriched in the pathways related to immune- and inflammation-linked diseases (e.g., *Staphylococcus aureus* infection, tuberculosis, and pertussis) and the immune-related pathways (e.g., nuclear factor-kappa B (NF-*κ*B) signaling pathway, complement and coagulation cascade, Toll-like receptor (TLR) signaling pathway, and NET formation) (Figure 5). The results of BPs and aberrant signaling pathways suggest that immune reactions and inflammation contribute to the progression of the carotid atherosclerotic plaques.

### 3.4. Identification and Validation of Expression Levels along with the Diagnostic Values of the Hub Genes

**Figure 6:**
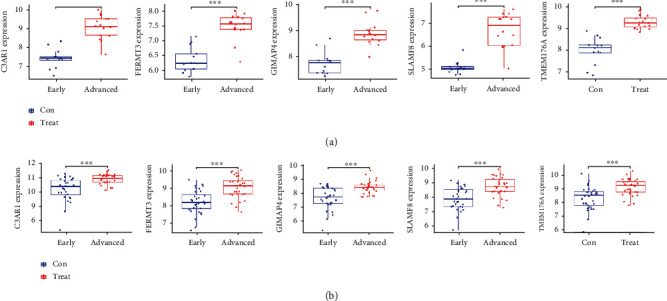
Validation of the expression levels of the identified hub genes. (a) Validating the hub gene expression in the GSE28829 dataset. *C3AR1, FERMT3, GIMAP4, SLAMF8,* and *TMEM176A* expression levels were remarkably elevated in the advanced carotid atherosclerotic plaques in relation to the early carotid atherosclerotic plaques. (b) Validating the expression of hub genes in the GSE43292 dataset. Similar findings were noted as those in the GSE28829 dataset.

The expression level of the 5 hub genes, namely *C3AR1*, *FERMT3*, *GIMAP4*, *SLAMF8*, and *TMEM176A*, were validated using box plots. In the GSE28829 dataset, the 5 hub genes showed a significantly lower expression in the early-stage carotid atherosclerotic plaques compared to the advanced-stage atherosclerotic plaques (Figure 6(a)). To improve the reliability of the results, we used an external dataset (GSE43292) for validating the expression level of the 5 hub genes. As shown in Figure 6(b), the 5 hub genes showed a significantly lower expression in the early-stage carotid atherosclerotic plaques compared to the advanced-stage carotid atherosclerotic plaques.

**Figure 7:**
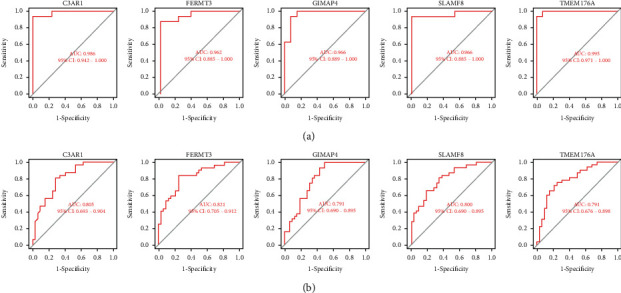
Validating the diagnostic performance of the hub genes. (a) Validating the diagnostic performance of the hub genes in the GSE28829 dataset. ROC curves and AUC values were used for assessing the ability to differentiate between the early- and advanced-stage carotid atherosclerotic plaques, with high specificity and sensitivity. (b) Validating the diagnostic efficiency of the hub genes in the GSE43292 dataset. Similar results were noted as those in the GSE28829 dataset. These findings prove that the 5 hub genes displayed an excellent diagnostic efficiency in the advanced-stage carotid atherosclerotic plaques. ROC: Receiver Operating Characteristic; AUC: Area Under the Curve.

Subsequently, the ROC curve analysis was carried out by comparing the values of the area under the curve (AUC) to determine the sensitivity and specificity of the hub genes in diagnosing the advanced-stage carotid atherosclerotic plaques. In the GSE28829 dataset, the AUC values of all five hub genes were > 0.95, indicating that these genes are of high value for the diagnosis of advanced-state carotid atherosclerotic plaques (Figure 7(a)). Their utility in clinical applications was verified by repeating the same ROC analysis using the above 5 hub genes in the GSE43292 dataset. Three of the hub genes showed AUC values of more than 0.80, while the *GIMAP4* and *TMEM176A* genes showed AUC values of 0.784 and 0.791, respectively (Figure 7(b)).

### 3.5. Infiltration Status of Different Immune Cells and Its Relationship with the Hub Gene Expression

**Figure 8:**
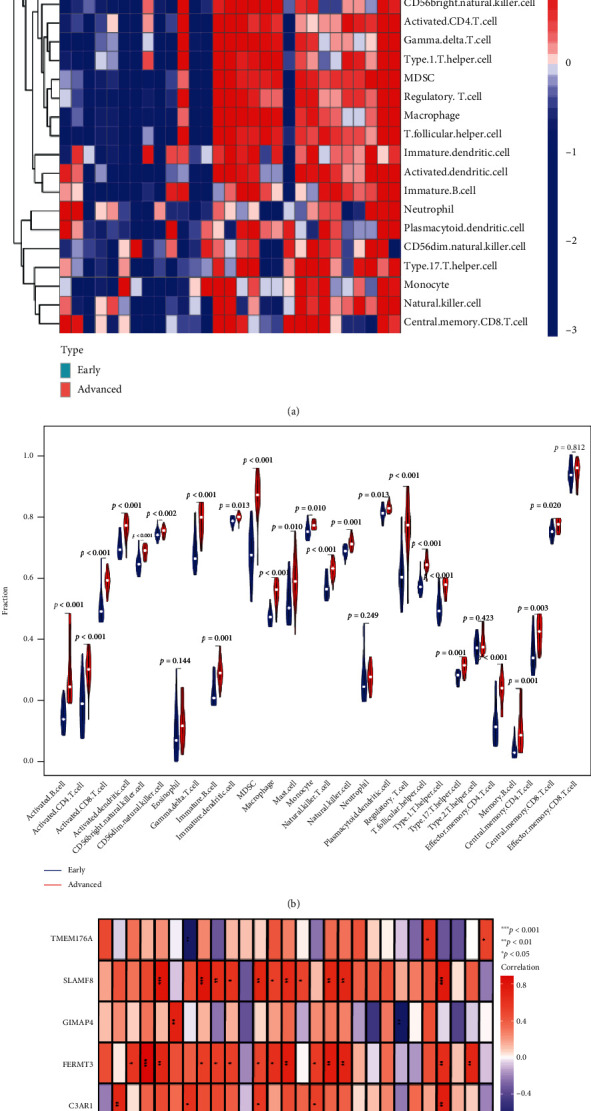
Analyzing the immune infiltration related to the advanced-stage carotid atherosclerotic plaques and the correlation analysis between 5 hub genes and immune cells. (a) Heat map and (b) violin plot highlighting the distribution of the 28 types of immune cells observed during the early- and advanced-stage carotid atherosclerotic plaques. (c) Relationship between the 5 hub genes and the infiltration levels of the immune cells.

Furthermore, the differences occurring in the infiltration of the immune cells between the advanced- and early-stage carotid atherosclerotic plaques were determined using the ssGSEA algorithm. The heat maps were used for observing the distribution of the 28 immune cells in the GSE28829 dataset (Figure 8(a)). Violin plots were constructed for presenting the immune cell infiltration analysis results. It was noted that the advanced-stage carotid atherosclerotic plaques showed a significantly higher infiltration of the immune cells like CD4^+^ T cells, CD8^+^ T, Tregs, monocytes, macrophages, T helper (Th), B cells, dendritic cells (DCs), and natural killer cells compared to the early-stage atherosclerotic plaques. These results suggested that these immune cells played a vital role in the advancement of the early-stage carotid atherosclerotic plaques to their advanced stage (Figure 8(b)). Thereafter, a correlation analysis was conducted between the hub genes and 28 types of immune cells, and the results revealed that the abundance levels of natural killer cells, Tregs, macrophages, mast cells, eosinophils, and gamma-delta T cells were positively related to the *FERMT3* and *SLAMF8* expression. On the other hand, the abundance of the activated DCs and myeloid-derived suppressor cells was positively related to the *C3AR1*, *FERMT3*, and *SLAMF8* expression. Furthermore, the abundance of neutrophils was seen to be negatively related to the *TMEM176A* expression, while it was positively related to the *C3AR1* expression. The *C3AR1* expression was positively related to the abundance of the type 17 Th cells and the immature B cells. Also, the abundance of the T follicular helper cells and the Type 1 Th (Th1) cells were positively related to the *FERMT3* expression (Figure 8(c)).

## 4. Discussion

High-throughput microarray technology has emerged recently as a fast and effective bioinformatics technique. It has offered a platform for disease diagnosis, treatment, and innovative medication discovery in addition to providing a framework for screening vital genes linked to the onset and progression of a variety of diseases. This article has been published as a preprint by our research team Zhang et al. [19]. This is the first study that has applied the LASSO technique for identifying the hub genes related to the progression of the carotid atherosclerotic plaques. 177 DEGs were identified in this report. According to the GO enrichment analysis, DEGs were predominantly enriched in processes related to leukocyte activation and migration, antigen processing and presentation, immunological response, and cytokine generation, all of which are linked to the development of the carotid atherosclerotic plaques [8, 20]. Higher levels of inflammatory cytokines (IL-1, TNF-*α*, and IL-6), adhesion molecules, and chemokines have been linked to the recruitment and infiltration of immune cells into the subendothelium, which results in plaque formation, rupture, and thrombus formation [8, 21, 22]. According to the analysis of KEGG signaling pathways, DEGs were mainly enriched in pathways linked to inflammatory diseases and the immune system (i.e., pathways related to NF-*κ*B signaling, TLR signaling, and NET formation). Given that NF-*κ*B plays a crucial regulatory function in immunity, apoptosis, stress responses, and cell differentiation, the NF-*κ*B signaling pathway has long been thought of as a classic proinflammatory signaling system [23, 24]. Earlier research has demonstrated that NF-*κ*B is activated in atherosclerotic lesions [25]. NF-*κ*B is a rapid response transcription factor that plays a role in immunological and inflammatory responses by stimulating the production of many immune cells like growth factors, chemokines, cytokines, cell adhesion molecules, and immunoreceptors [26]. These gene products trigger an immune-inflammatory response that damages vascular walls and affects vascular cell function, resulting in the progression of arteriosclerotic plaques [27]. Recent research has shown that inhibiting the NF-B signaling system can lessen the inflammatory burden and could be a promising antiatherosclerotic, anti-inflammatory, antiangiogenic, and antiapoptotic therapeutic target [28, 29].

TLRs, described as established pattern recognition receptors in the immune system, have the ability to recognize pathogen-associated molecular patterns expressed by a variety of infectious agents and provide a strong link between local innate and adaptive immunity [30]. When these receptors get activated, they trigger the intracellular signaling cascade that is MyD88 or TRIF-mediated, which eventually stimulates the production of the pro- and anti-inflammatory cytokines [31]. TLRs transmit activating signals to secrete proinflammatory cytokines in large quantities, thus inducing the transitional activation of inflammation, which can play a vital role in the advancement of early-stage plaques to their advanced stages [32]. Large, extracellular, web-like structures known as NETs are primarily released through a process of neutrophil cell death known as NETosis to trap and kill microbes in particular physiological conditions; NETs are composed of extracellular strands of decondensed DNA in complex with granule proteins and histones [33, 34]. Pathologically speaking, NETs can, however, increase the inflammatory response by inducing the activation of inflammatory factors and cells, which promotes the progression of plaques from their early stage to advanced stages [35, 36]. Additionally, the interaction between the NF-*κ*B signaling pathway and NETs aggravated atherosclerosis [37]. The results of the DEG enrichment analysis and the findings from these investigations were in good agreement, indicating that DEGs were primarily responsible for the advancement of the early-stage carotid atherosclerotic plaques to their advanced stage.

WGCNA and LASSO, which are widely used as common methods of bioinformatic analysis, were used for avoiding the drawbacks of the conventional DEG-based screening techniques [38] and improving the accuracy of the screening of target feature-linked genes [39]. The genes whose expression was highly correlated with advanced-stage carotid atherosclerotic plaques identified using WGCNA were matched with previously identified DEGs to identify genes with both differential expression and correlation. Five hub genes, i.e., *C3AR1*, *FERMT3*, *GIMAP4*, *SLAMF8*, and *TMEM176A*, were eventually screened via LASSO. The expression levels of the above 5 hub genes showed a significant difference between the early-stage and the advanced-stage carotid atherosclerotic plaques and were validated in an external dataset. Specifically, these five genes showed significantly high expression and good diagnostic efficacy in advanced-stage carotid atherosclerotic plaques.

*C3AR1* encodes for an orphan G protein-coupled receptor for the C3a. The gene product can induce inflammation by binding to complement C3a [40]. *C3AR1* has a crucial role in TLR activation in innate DCs and influences effector T cell responses [41]. The expression of *C3AR1* was higher in carotid plaques than in control arteries [42]. This suggests that *C3AR1* overexpression is involved not only in disease onset but also in disease progression. *FERMT3* encodes for important cytoplasmic proteins that are needed for platelet aggregation, leukocyte transmigration, integrin activation, and thrombosis [43, 44]. *FERMT3* overexpression suppressed NF-*κ*B activation and induced apoptosis [45]. The activation of the integrin-mediated platelet aggregation and adhesion triggered arterial thrombosis and several cardiovascular events [46]. Platelets are seen to play a crucial role in the promotion of inflammation and foam cell formation, leukocyte adhesion, and transmigration in the vessel wall, which, in turn, promoted the carotid atherosclerotic plaque progression [47, 48]. *FERMT3* was upregulated in arterial plaques compared with that in healthy controls, and confocal immunofluorescence analysis showed the colocalization of *FERMT3* with CD68-positive cells [49]. This is in good agreement with the correlation analysis data for the expression of the 5 hub genes within the immune cells. These data indicate that FERMT3, as an important modulator of integrin-mediated mechanisms, participated in the advancement of the carotid atherosclerotic plaques. Proteins of the GIMAP family were deferentially regulated during the human Th cell differentiation and were associated with immune-mediated disorders in an earlier animal study [50]. *GIMAP4* was upregulated by IL-12 and other Th1 differentiation-inducing cytokines in cells differentiating toward a Th1 lineage and downregulated by IL-4 in cells differentiating toward a Th2 [51]. *GIMAP4* could accelerate T-cell apoptosis induced by caspase-3 activation and phosphatidylserine exposure, which contributed to the Th-cell subtype-triggered immunological balance [52]. As demonstrated by our results, *GIMAP4* expression showed a negative correlation with CD4^+^T cell abundance. The *SLAM* family (*SLAMF*) comprises a group of nine structurally related hematopoietic cell-specific receptors that are differentially expressed and play different roles in many immune cells [53]. *SLAMF8*, a nonclassical *SLAMF* member, does not include signaling motifs in its short cytoplasmic tails, in contrast with normal *SLAMF* receptors. In the past, researchers have shown that a combined *SLAMF8* deficiency suppressed the inflammatory responses via the mechanism of downregulating the expression of TLR4 on the macrophages [54, 55]. A deletion in *SLAMF8* significantly inhibited TLR4 upregulation and NF-*κ*B activation [55]. *TMEM176A* is involved in tumor development [56, 57]. *TMEM176A* inhibits the maturation and activation of DCs to modulate DC function, thereby influencing the development of innate and adaptive immunity [58, 59]. Based on these results, *C3AR1* and *FERMT3* are significantly involved in the development of the carotid atherosclerotic plaques. Although *GIMAP4*, *SLAM8*, and *TMEM176A* are closely associated with inflammatory responses, their exact roles in the advancement of the carotid atherosclerotic plaques are not clear and need to be investigated further.

Furthermore, the differences noted in the immune cell infiltration between the advanced- and early-stage carotid atherosclerotic plaques were determined using the ssGSEA algorithm. It was noted that the advanced-stage carotid atherosclerotic plaques showed a significantly higher infiltration level of the immune cells like CD8^+^ T, CD4^+^ T cells, Tregs, monocytes, macrophages, T helper (Th) cells, and DCs compared to the early-stage, atherosclerotic plaques. Th17 cells are derived from CD4^+^ T cells, and they got their name because they can secrete high levels of IL-17 [60]. Th17 cells promote plaque fibrosis, whereas Th1 cells promote the formation of atherosclerotic plaques [61]. Tregs are often regarded to exert a protective function in atherosclerotic plaque formation [62]. However, Tregs show the loss of *FOXP3* expression and immunosuppressive function during atherosclerosis progression, owing to which a fraction of these cells is transformed into follicular Th cells, which are proatherogenic [63]. These findings illustrated the essential role played by the T-cell immune homeostasis disruptions in the development of carotid atherosclerotic plaques.

## 5. Conclusions

The WGCNA and LASSO techniques were implemented for screening five hub genes (C3AR1, FERMT3, GIMAP, SLAM8, and TMEM176A) and one hub module (brown module) that may be implicated in the progression of the carotid atherosclerotic plaques. Then, bioinformatics analysis was carried out using ssGSEA to investigate the difference in the immune cell infiltration status between the early- and advanced-stage carotid atherosclerotic plaques. These results offer insightful information regarding the infiltration level of the immune cells during the progression of the carotid atherosclerotic plaques. In addition, the identification and validation of hub genes provide evidence for further research on the pathogenesis and early diagnosis of and targeted drug intervention for carotid atherosclerotic plaques. However, these conclusions have not been confirmed by experiments. Further experiments to elucidate the detailed biological functions of these genes are the focus of future research.

## Data Availability

The data are public and can be downloaded from the GEO database.
